# Dialysis Disequilibrium Syndrome Induced Seizure Following Hemodialysis

**DOI:** 10.7759/cureus.17821

**Published:** 2021-09-08

**Authors:** Chandra Wirawan, I Ketut Sumada, Desie Yuliani, Ni Ketut Candra Wiratmi, Riki Sukiandra

**Affiliations:** 1 Department of Neurology, Wangaya General Hospital, Bali, IDN; 2 Department of Neurology, Arifin Achmad General Hospital, Riau, IDN

**Keywords:** brain edema, chronic kidney insufficiency, dialysis, seizures, urea

## Abstract

Dialysis Disequilibrium Syndrome (DDS) is a set of neurological signs and symptoms that can occur during or following dialysis. Osmotic fluid gradient alteration caused by the dialysis process can cause cerebral edema. This process relates to the changes of high gradient urea in both the blood and central nervous system (CNS), which can modulate extracellular fluid influx into brain cells. A 77-year-old woman presented with chronic kidney disease (CKD) and hypertension with headache and tonic-clonic seizure following initial hemodialysis. In this case, we identified that adjustment to the hemodialysis prescriptions such as duration, blood flow rate, and target reduction of blood urea would be the key to avoid seizure following hemodialysis.

## Introduction

The number of chronic kidney disease (CKD) patients who undergo hemodialysis is increasing during these years. In 2012, approximately 19,621 CKD patients had their first hemodialysis and five years later, this number had become 30,831. The highest prevalence of CKD (30.45%) is seen in the middle-aged group. About 90% of hemodialysis patients were CKD stage V [1]. Approximately 50 million people have experienced seizures worldwide and 8.8% of seizures are due to hemodialysis [2]. The prevalence of seizures was approximately eight times higher in hemodialysis patients than in the common population [3]. Patients undergoing their first hemodialysis are at a higher risk to have dialysis disequilibrium syndrome (DDS). Neurological symptoms and signs during or shortly following dialysis are known as DDS. Seizure after hemodialysis is associated with the osmotic fluid shift due to the alteration of urea level [4,5]. Although the prevalence of seizures in hemodialysis was quite common, the prevalence of seizures due to DDS was quite rare [6]. Seizure is the most common sign in DDS. Other manifestations of DDS are alteration in mental status, asterixis, coma, and death [4]. The mortality rate of hemodialysis patients with a history of seizure is higher than those without such a history (31.9% vs 23.3%) [6]. This report presents the case of a patient with CKD stage V who had seizures after initial hemodialysis.

## Case presentation

A 77-year-old woman with anemia, CKD stage V, and hypertension was transferred from a medical clinic to the emergency department of Wangaya General Hospital, Bali. The patient had nausea and vomiting with a frequency of more than ten times a day for the past ten days. Fatigue and a decrease in appetite were also found. The patient had hypertension for five years with uncontrolled blood pressure and did not consume any drugs. There was no previous history of seizure and hemodialysis.

On arrival, she had a Glasgow Coma Scale (GCS) of E4V5M6, blood pressure was 160/70 mmHg, heart rate was 69 beats per minute, respiratory rate was 20 breaths per minute, body temperature was 36.5ºC, and oxygen saturation was 99% in room air. The other examinations including respiratory, cardiovascular, abdominal, and neurological were normal. The result of laboratory blood test revealed haemoglobin 10.7 g/dL (12-16 g/dL), blood glucose 120 mg/dL (80-200 mg/dL), blood urea nitrogen (BUN) 179 mg/dL (10-50 mg/dL), serum creatinine 11.6 mg/dL (0.3-1.2 mg/dL), serum sodium 116 mmol/L (130-145 mmol/L), potassium 6.3 mmol/L (3.5-5.5 mmol/L), and chloride 103 mmol/L (95-108 mmol/L). According to the Cockcroft-Gault equation, the calculated glomerular filtration rate (GFR) was less than 15 mL/minute/1.73 m².

**Figure 1:**
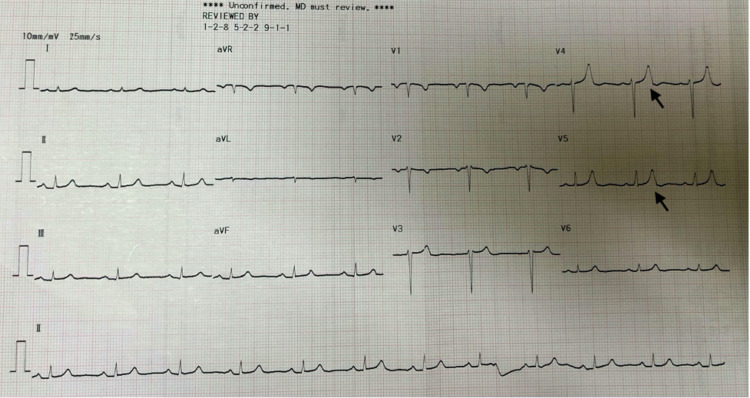
ECG showing normal sinus rhythm with peaked T-waves

The patient was admitted for the management of CKD stage V, anemia, hypertension, and electrolyte imbalance. Her initial ECG showed normal sinus rhythm with peaked T waves (Figure 1). During hospitalization, no seizure episodes were reported. Subsequently, hemodialysis was planned for the next two days as the patient were on the waiting list. The first hemodialysis was performed after two days, which lasted for three hours with a blood flow rate of 300 mL/ per minute, and 1000ml of total body fluid was removed. The patient suffered an episode of first witnessed tonic-clonic seizure approximately seven hours following hemodialysis. This seizure episode was resolved by itself, and the patient was back to baseline with minor complaints of headache following dialysis.

**Figure 2:**
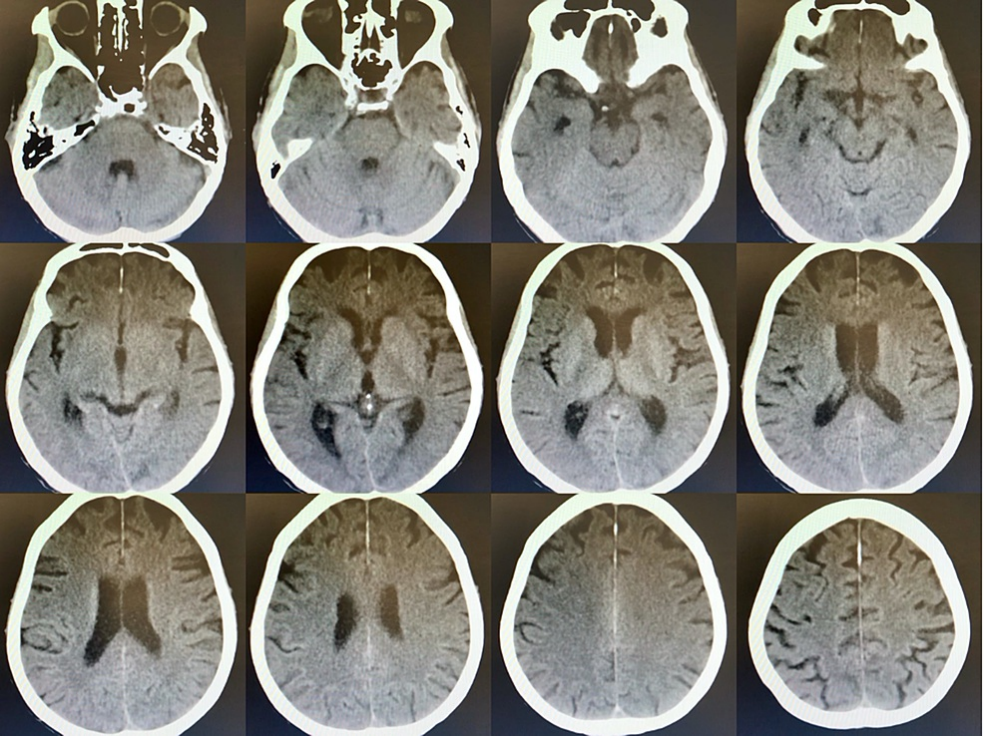
Non-contrast CT brain (after the seizure episode following the first hemodialysis) did not show any hemorrhage, infarct, and space-occupying lesion

After a neurologic consult, a non-contrast CT brain (Figure 2) was obtained, which did not show any hemorrhage, infarct, or space-occupying lesion (SOL) intracranially. Neurology also recommended diazepam 10 mg IV (as needed) for any future breakthrough seizure episodes. Blood chemistry after initial hemodialysis revealed serum sodium at 134 mmol/L, potassium at 4.5 mmol/L, chloride at 98 mmol/L, BUN at 132 mg/dL, and serum creatinine at 11.1 mg/dL. The second hemodialysis was completed three days after the first session with a similar blood flow rate of 300 mL/min, removal of 1000 ml total body fluid in four hours duration. The patient suffered another tonic-clonic seizure eight hours after the hemodialysis lasting thirty seconds and resolved by itself before diazepam was injected. A re-evaluation of blood chemistry was done. Blood electrolyte was normal, BUN was 95 mg/dL, and serum creatinine was 3 mg/dL. In the next four days, the patient had received the third session of hemodialysis with adjusted duration and blood flow rate (two hours; 150 mL/min). This time, the patient did not experience any seizure activity at all. She was diagnosed with dialysis disequilibrium syndrome.

## Discussion

DDS comprises neurological symptoms with varying severity that affects dialysis patients. The risk factors of DDS are first dialysis treatment, older age, hypertension, CKD, hyperglycemia, hypernatremia, hyperuricemia, metabolic acidosis, pre-existing cerebral edema, and neurological abnormalities, and any conditions that can increase the permeability of the blood-brain barrier, such as infection (meningitis, vasculitis), tumors of CNS, hemolytic uremic syndrome, and thrombotic thrombocytopenic purpura. The symptoms of DDS relate to osmotic fluid shift, which can be affected by urea and dialysis [4,7]. Although the pathogenesis of DDS is unknown, there are three theories that can explain the syndrome: reserve osmotic shift, intracerebral acidosis, and idiogenic osmole. Reserve osmotic shift theory is frequently used to explain the pathogenesis of DDS. This theory explains that the reduction of blood urea caused by hemodialysis leads to a transient osmotic gradient. Therefore, the fluid can influx to brain cells from the extracellular fluid, which can promote cerebral edema. In animal studies, urea transporters (UTs) and aquaporins (AQPs) manage osmolality in the brain. UT-B1 (UTs in brain) declines while AQP4 and AQP9 elevate. This process results in fluid transfer from extracellular to brain cells in animal studies [4,8]. Dialysate, which contains bicarbonate in the dialysis process, is used to fix metabolic acidosis. Thus, it can increase the blood pH level. As compensatory to this process, hyperventilation occurs and converts bicarbonate into carbon dioxide (CO_2_). Carbonic acid exists in the brain due to the rapid diffusion of CO_2_ to the brain. This process decreases the pH level in cerebrospinal fluid (CSF), which can increase intracellular osmolality and promotes cerebral edema. The idiogenic osmole theory defines the production of idiogenic osmoles as an adaptive response from the cerebral cortex in 48 hours that occurs when there is a rapid correction of hyperglycemia or hypernatremia. Idiogenic osmoles mediate the gradient osmotic, which favors water movement intracellularly and causes cerebral edema [4,7-9].

The clinical manifestations of DDS are nausea, vomiting, headache, dizziness, muscle spasm, confusion, tremors, visual disturbances, asterixis, seizure, coma, and death. The symptoms of DDS appear during or several hours following hemodialysis. No abnormal finding can be observed in neurologic examination [4]. Consistently, our patient experienced the symptoms of DDS such as headache and seizure after hemodialysis. Normal neurological examination was also observed in this patient. As mentioned, our patient also had risk factors of DDS such as first dialysis treatment, older age, CKD, hypertension, and hyperuricemia. Imaging studies like non-contrast CT scan brain should be performed to exclude the causes of seizure, such as intracerebral hemorrhage, cerebral infarction, and SOL. Some cerebral edema can be found from head CT-scan in DDS in acute settings [8]. Electroencephalography (EEG) and MRI are not routinely done in DDS due to unspecific features of DDS in EEG and MRI. Slowing activity and increasing relative power in the delta frequency band in EEG are found in hemodialysis patients [10]. Our patient had no cerebral edema in the head CT scan because it was not performed in an acute setting, one day after the first seizure, due to some technical difficulty. It is also possible that cerebral edema might have resolved by the time the CT was completed. Nevertheless, a head CT scan should be performed to exclude other differentials. Brain atrophy was found in this patient most likely due to aging. MRI and EEG were not done as they did not contribute to diagnosing DDS.

There is no specific treatment of DDS. Hyperosmolar solution (mannitol or glycerol) is not effective because DDS is a self-terminating syndrome. No anti-epileptic is needed for prevention of seizures in DSS. Prevention is more important than treatment. Management of risk factors and setting, both duration and blood flow rate of hemodialysis, plays an important role in the prevention of DDS. Although there is no certainty about hemodialysis prescription, many experts suggest having two hours of hemodialysis and a slower blood flow rate (150-250 mL/min) with up to 40% reduction in blood urea as target dialysis. Hemodialysis should be stopped immediately with the notice of any signs of DDS [4,6-8,11].

In this case, the patient had hemodialysis and laboratory blood test, included BUN and serum creatine. The reduction of BUN in every hemodialysis was around 26-28%. However, the first and second hemodialysis had higher blood flow rate and longer duration than suggested (300 mL/min vs 150-250 mL/min; three to four hours vs two hours). When the patient had the third hemodialysis with a slower blood flow rate (150 mL/min) and shorter duration (two hours), there was no seizure reported. This might be indicative of management with blood flow rate and duration as the key to avoid episodes of DDS besides also reducing BUN level appropriately.

## Conclusions

This case reported a patient with many risk factors developing DDS after initial hemodialysis. Diagnosis of DDS was made by clinical history and manifestations, blood laboratory tests (especially renal function tests, BUN, and serum creatine), and imaging studies. A Head CT scan should be done to rule out other diagnoses in seizures following hemodialysis. Even though DDS is a self-terminating syndrome, about 8.8% of seizures are associated with hemodialysis. Given it is a complex presentation and there is a lack of awareness among physicians, DDS should be considered early on during evaluation. Prevention of DDS with adjustment of duration, blood flow rate, and target reduction of blood urea is substantial and helped our patient to not have any further seizure episodes or symptoms of DDS after hemodialysis. However, this prescription should be further evaluated to have a defined cut-off point to prevent DDS.
